# PERCEPTIONS OF EXERGAMES FOR FALLS PREVENTION AMONG SENIORS AND THERAPISTS IN ASSISTED LIVING FACILITIES

**DOI:** 10.1093/geroni/igz038.231

**Published:** 2019-11-08

**Authors:** Emma K Stanmore, Lex D de jong, Dawn A Skelton, Wytske Meekes, Alexandra Mavroeidi, Chris Todd

**Affiliations:** 1 University of Manchester, Manchester, United Kingdom; 2 Curtin University, Perth, Australia; 3 Glasgow Caledonian University, Glasgow, United Kingdom; 4 University of Tilburg, Tilburg, Netherlands; 5 University of Strathclyde, Glasgow, United Kingdom

## Abstract

Strength and balance exercise programmes are effective in reducing the rate and risk of falls but uptake and adherence to community based programmes are low. Exergaming technology may be a promising tool to increase exercise uptake and adherence among seniors due to their motivational features. As yet, there is little published on the views of older people and therapists of their experiences of using Exergames to reduce falls in assisted living facilities that may inform implementation. This study is the qualitative component of a cluster randomized controlled trial that investigated the use of purpose-built strength and balance Exergames in community-dwelling older people in 18 assisted-living facilities in the UK. Data collection included 36 hours’ observation, semi-structured interviews with 5 physiotherapists, assistants and researchers, and 13 focus groups with residents of assisted living facilities who had participated in a 12 week Exergame programme. Transcribed data were analysed using thematic content analysis to identify themes arising from older users’ and therapists’ experiences of using the Exergames. Findings suggest that the senior users enjoyed using the strength and balance Exergames and reported physical, psychological and social benefits. Although some games were generally favoured, there was no overall consensus on game preferences although social components, feedback, music and colourful animation appeared to increase the appeal of the Exergames. This is the first study exploring older users and therapists’ perceptions of exergames in assisted living facilities and provides valuable insight into the barriers, facilitators, contextual factors and perceived benefits or drawbacks following 12 weeks use.

